# Evaluating phase separation in live cells: diagnosis, caveats, and functional consequences

**DOI:** 10.1101/gad.331520.119

**Published:** 2019-12-01

**Authors:** David T. McSwiggen, Mustafa Mir, Xavier Darzacq, Robert Tjian

**Affiliations:** 1 Department of Molecular and Cell Biology, University of California Berkeley, California 94720, USA;; 2 California Institute of Regenerative Medicine Center of Excellence, University of California Berkeley, California 94720, USA;; 3 Howard Hughes Medical Institute, University of California Berkeley, California 94720, USA

**Keywords:** fluorescence recovery after photobleaching, condensate, liquid–liquid phase separation, phase separation

## Abstract

The idea that liquid–liquid phase separation (LLPS) may be a general mechanism by which molecules in the complex cellular milieu may self-organize has generated much excitement and fervor in the cell biology community. While this concept is not new, its rise to preeminence has resulted in renewed interest in the mechanisms that shape and drive diverse cellular self-assembly processes from gene expression to cell division to stress responses. In vitro biochemical data have been instrumental in deriving some of the fundamental principles and molecular grammar by which biological molecules may phase separate, and the molecular basis of these interactions. Definitive evidence is lacking as to whether the same principles apply in the physiological environment inside living cells. In this Perspective, we analyze the evidence supporting phase separation in vivo across multiple cellular processes. We find that the evidence for in vivo LLPS is often phenomenological and inadequate to discriminate between phase separation and other possible mechanisms. Moreover, the causal relationship and functional consequences of LLPS in vivo are even more elusive. We underscore the importance of performing quantitative measurements on proteins in their endogenous state and physiological abundance, as well as make recommendations for experiments that may yield more conclusive results.

## Abstract (precis)

In this Perspective, McSwiggen et al. analyze the evidence for liquid–liquid phase separation (LLPS) condensates across multiple cellular processes. They find that the evidence for in vivo LLPS is often inadequate to discriminate between phase separation and other possible mechanisms, and urge the application of more stringent criteria and more appropriate experimental approaches to understand the functional role of LLPS condensates in cellular organization.

Fundamentally, a cell is a collection of molecules compartmentalized in a manner to modulate biochemical reactions that support diverse cellular activities. The challenges faced by a cell in managing these biochemical processes scales with organismal complexity. In eukaryotes, where some cellular tasks can require the coordinated activity of tens to hundreds of individual molecular components, elaborate mechanisms have evolved to ensure that these assemblies occur, and furthermore, that they do so on timescales relevant to their biological function. Prototypical examples of cellular organization are the membrane-bound organelles, but it has long been appreciated that many compartments exist in the cell without an enclosing membrane (Montgomery 1898; Wilson 1899).

In the past decade, a fresh perspective on membraneless compartments—now often referred to as biomolecular condensates (Banani et al. 2017)—has led to a resurgence in the idea that a majority of these compartments may exist as separate liquid phases (Courchaine et al. 2016). There has been a renaissance in understanding how liquid–liquid phase separation (LLPS) might function in compartment formation and maintenance (Hyman et al. 2014; Banani et al. 2017). Perhaps the most often cited example is the nucleolus, where a convergence of studies examining its liquid-like behavior (Brangwynne et al. 2011), supported with biochemical (Feric et al. 2016; Mitrea et al. 2016) and in vivo experiments (Berry et al. 2015; Weber and Brangwynne 2015), collectively support a model where the nucleolus behaves as a separate liquid phase within the nucleus. Inspired by these and other early examples of compartments with liquid-like properties (Brangwynne et al. 2009), there has been a surge of publications revisiting the formation of well-known cellular compartments through the lens of LLPS. Far from being the peculiarity it once was, phase separation now has become, for many, the default explanation to rationalize the remarkable way in which a cell achieves various types of compartmentalization, prompting significant debate within the scientific community (Mir et al. 2019).

Much of the debate around LLPS condensates arises because it is unclear how strong the evidence for in vivo LLPS is, particularly when LLPS is invoked so broadly across many cellular contexts. The current focus on LLPS as a mechanism may come at the expense of understanding alternative mechanisms by which a high local concentration of factors can be achieved in the absence of a membrane. For example, while nucleoli exhibit many properties consistent with LLPS, the formation of nucleoli and many other nuclear bodies have previously been explained by alternative mechanisms (Mao et al. 2011a,b; Shevtsov and Dundr 2011). In a recent study we found that Herpes Simplex Virus replication compartments derive their ability to concentrate cellular factors through transient nonspecific binding to the viral DNA in a manner distinct from liquid–liquid phase separation (McSwiggen et al. 2019). Despite this mechanistic distinction, these replication compartments display many of the hallmarks that are often deemed sufficient to claim that such a compartment is formed via LLPS (McSwiggen et al. 2019).

Our data on replication compartments, as well as other recent studies from our group (Mir et al. 2017, 2018; Chong et al. 2018) demonstrate that there are multiple routes to establish regions with high local concentrations of specific factors inside the cell. These studies prompted us to critically reexamine the current evidence for LLPS in vivo. The appeal for invoking phase separation is understandable, as it presents a way to rationalize—and at least superficially explain—certain behaviors of cellular compartments. However, in light of various recent studies and upon further analysis, we find that the evidence for LLPS occurring in the cell is often far from conclusive. This is not to imply that LLPS cannot function in biological contexts, but rather to highlight how the tests commonly used in probing LLPS are insufficient to rule out other mechanistic interpretations.

In this Perspective, we summarize the evidence used to diagnose liquid–liquid phase separation in vivo. Recently, others have similarly urged caution in overinterpreting in vivo experiments to test LLPS (Alberti et al. 2019), but the issues in this field run deeper than the authors discuss. This Perspective is, to our knowledge, the first to systematically and holistically consider the evidence presented by this emerging field. We first provide a summary of the state of evidence for LLPS condensates across multiple contexts, and address important considerations for this evidence. Second, we address the evidence for the functional consequences of LLPS in the underlying biological processes being studied. Finally, we urge the application of more stringent criteria and more appropriate experimental approaches to understand the functional role of LLPS condensates in cellular organization.

## A diagnostic problem

**Figure 1.**
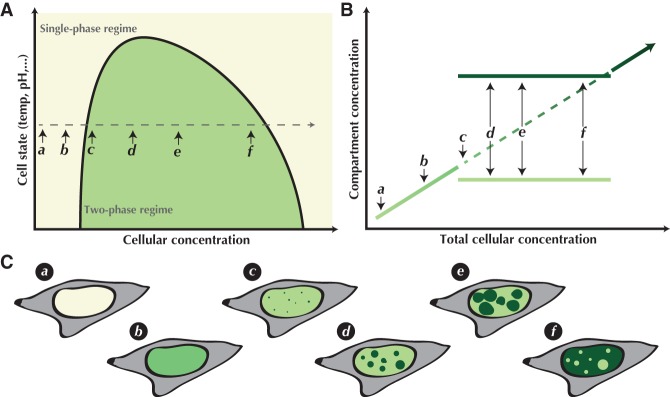
Liquid–liquid phase separation is a function of concentration. (*A*) A schematic of a phase diagram depicting under what set of environmental conditions (temperature, salt concentration, pH, etc.) the system will remain as a single phase or spontaneously form two phases. An increase in the *y*-axis would represent any environmental change that would weaken monomer interactions, e.g., increasing temperature. The dashed line depicts how the system responds to increasing protein concentration, further illustrated in *B* and *C*. (*B*) For proteins that can phase separate, at a certain critical concentration (c), droplets form. Past this critical concentration, production of more protein increases droplet size but does not change the concentrations in either phase, until eventually the concentrated phase entirely fills the space whereupon the system returns to the one-phase regime (*A*). (*C*) An illustration of the processes depicted in *A* and *B* as it occurs in the cell—in this hypothetical example, in the nucleus.

Phase separation arises as a result of supersaturation. When a molecular species is at or above a critical concentration based on the specific cellular conditions (temperature, pH, etc.), it becomes more thermodynamically favorable to partition into a high-concentration phase and a low-concentration phase (Banani et al. 2017). Production of more of the protein in a two-phase regime does not increase the protein concentration in either of the phases, but rather results in changes in the relative volumes occupied by the two phases (Fig. 1). A simplistic example of this is the nucleation and growth of water droplets on a cold glass. Accumulating evidence suggests the potential for LLPS to occur widely with biological macromolecules as well, and it has been shown that certain classes of proteins—as well as RNA and other biological polymers—readily undergo LLPS in vitro (Jain and Vale 2017; Wang et al. 2018).

The topic of phase separation in biology has been extensively reviewed elsewhere, and the reader is encouraged to refer to these reviews for a more thorough explanation of the forces that drive liquid–liquid demixing (Hyman et al. 2014; Brangwynne et al. 2015; Banani et al. 2017; Boeynaems et al. 2018). Much of what we know now has foundations in early works on polymer physics (Overbeek and Voorn 1957) and has been advanced by efforts to improve crystallographic methods for which phase separation was used as a means of increasing a protein's concentration without it crashing out of solution (Lomakin et al. 1996; Asherie 2004; Vekilov 2010). Other types of phase transition have also been proposed to occur in cells. For example, it has been proposed that some proteins may transition into gel-like structures (Kato et al. 2012; Kwon et al. 2013) or liquid-crystalline structures (Rog et al. 2017), again drawing models from lessons learned in polymer physics and materials science for inspiration.

Physical models exist to explain liquid demixing (Lomakin et al. 1996; Velasco et al. 1998), and for purified components like proteins or nucleic acids, there exist rigorous standards by which one may determine whether a given system is undergoing liquid–liquid demixing. Modulating the concentration of a polymer, the ionic strength of the buffer, the temperature of the system, and intra- or interpolymer interactions can all quantifiably change the propensity of the polymer to demix (Lomakin et al. 1996; Velasco et al. 1998; Vekilov 2010; Brangwynne 2013). Following this model, beautiful in vitro experiments have been performed demonstrating the ability of LLPS systems to exhibit exclusivity (Nott et al. 2015; Banani et al. 2016; Feric et al. 2016); to form and dissolve on the basis of post-translational modifications (Li et al. 2012; Lu et al. 2018) and to exhibit changes in viscosity and to “ripen” or harden over time (Patel et al. 2015; Wegmann et al. 2018).

These studies suggest that, at least in vitro, LLPS is particularly pervasive for proteins containing large disordered and low-complexity domains that enable multivalent homo- and heterotypic protein–protein interactions. While elegant biochemical experiments have provided essential insights into the physical properties of macromolecules that undergo LLPS, it remains less clear to what extent LLPS is happening in the crowded milieu of the cell. The intracellular environment itself is immensely more complex by virtue of the coexistence of hundreds of thousands of other macromolecular and small-molecules species that share the same solvent in a highly confined volume. It remains an open question to what extent the physical models built on in vitro studies hold true when dealing with the innumerable possible homo- and heterotypic interactions inside the cell, each of which has the potential to facilitate or antagonize LLPS or molecular function.

In vivo, there is often much less control over the various parameters that should ideally be altered to test for LLPS to discriminate between it and other potential mechanisms. For example, while it is possible to tune, to a limited degree, parameters like the concentration of a few target proteins or the ionic strength of the solution, additional nontrivial controls are required to ensure that the resulting changes are not due to secondary effects as the cell responds to a changing environment.

## An accumulation of qualitative evidence

The challenges of modulating parameters critical to validate phase transitions in vivo have led researchers to instead rely heavily on descriptive characteristics. Roundness as a proxy for surface tension, the ability to undergo fusion or fission, changes in refractive index, and dynamic rearrangement within the phase as measured by FRAP are all routinely used to diagnose LLPS in vivo, largely based on the observation that in vitro droplets display these same liquid-like behaviors. We examined 33 studies, collectively making claims for 50 examples of in vivo LLPS for a range of cellular systems and organisms (Table 1). Without drawing any specific conclusions regarding the quality of the data in a given study, we categorized evidence based on whether the study used qualitative descriptors (+) or quantitative measurements (+++) to assess a given criterion. For example, a study reporting that “the droplets were round and could be seen to “fuse” received a “+” for the “roundness” and “fusion/ripening” criteria, whereas a study that quantifies the degree of roundness or conservation of material after fusion received a “+++”. If a criterion is not mentioned, or if the assay does not apply to the system under study, it received a “−”, and if it cites other literature that previously reported the claim, it received a “PR.”

**Table 1.**
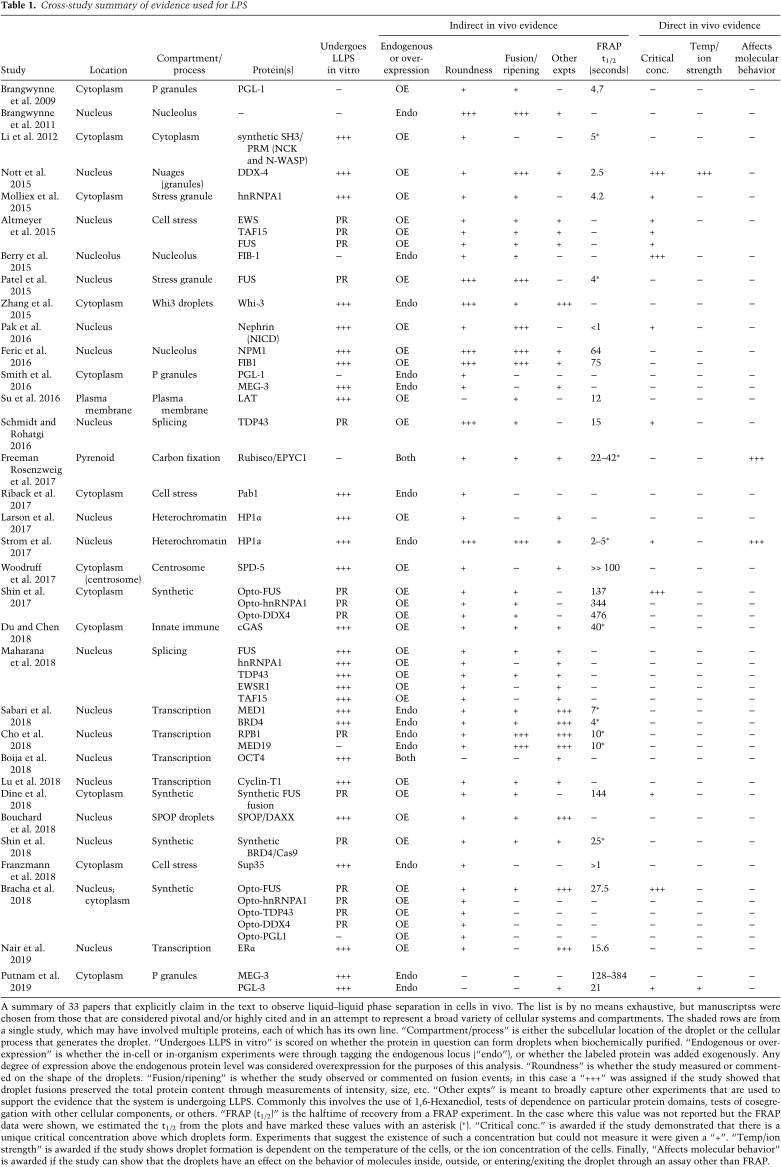
Cross-study summary of evidence used for LPS

**Figure 2.**
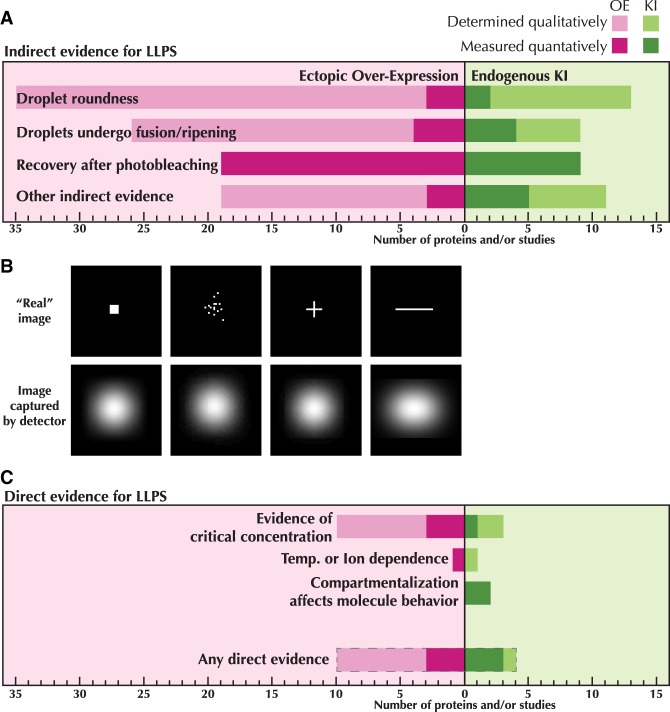
Evidence for LLPS in cells is largely phenomenological. (*A*) A bar graph quantifying the use of descriptive or phenomenological criteria in the studies from Table 1, separated into experiments that are performed on the endogenous protein (knock-in, KI) compared with those in overexpression systems (OE). The *x*-axis is the number of proteins from the 33 studies that were claimed to display that evidence. (*B*) A simulated example of how diffraction-limited fluorescence imaging can obscure fine features. The *top* row depicts various simulated structures, and the *bottom* row is the image acquired by the microscope detector. (*C*) A bar graph quantifying the use of assays which give direct evidence for LLPS in vivo. “Any direct evidence” is any example which demonstrated at least one of the categories of direct evidence. See Table 1.

As LLPS is critically dependent on concentration, a crucial test to determine whether it is occurring is to identify a critical concentration above which droplets exist and below which they do not (Asherie 2004). Despite this critical dependence, the majority of the studies we examined performed all of their in vivo measurements using ectopic overexpression (Fig. 2). The degree of ectopic expression varies case-by-case, but many multi-phase systems are exquisitely sensitive to changes in concentration (indeed, this fact is often used to support the biological function of LLPS) (Alberti et al. 2019). Furthermore, at least in some cases, it has been suggested that cellular systems exist just on the cusp of a two-phase regime, in which case even the mildest overexpression could dramatically influence the outcome and interpretation of the data (Narayanan et al. 2019). Such overexpression introduces significant caveats into the conclusions that can be made from these studies.

Another surprising feature that stands out when examining the evidence in these studies is how heavily they rely on the descriptive properties of LLPS, rather than on quantitative tests. A majority of the cases reported roundness and observations of puncta fusion (48 and 35 out of 50 proteins, respectively), but the evidence provided is often a single image or movie, whereas few (six and eight examples out of 50, respectively) measure these behaviors in a quantitative manner (Fig. 2). Furthermore, with the exception of fluorescence recovery after photobleaching (FRAP) experiments—which have their own limits to be discussed in the next section—many studies only use qualitative observations or other indirect lines of evidence for LLPS in vivo.

One of the major considerations with these indirect measurements, particularly with roundness and fusion, is the fact that diffraction-limited features have a tendency to look round and can obscure the true underlying structure. This is especially true if the size of the features is near to, or below, the resolution of the system (Fig. 2B), as is the case for many putative LLPS systems (Boija et al. 2018; Cho et al. 2018; Maharana et al. 2018; Sabari et al. 2018; Guo et al. 2019). These incipient attempts at characterizing putative LLPS condensates in vivo using various imaging modalities show promise, but also underscore a potential confounding challenge. Roundness, for example, can be measured but is often provided only as a snapshot to judge by. This is exacerbated by the post-processing and image representation methods that may or may not have adhered to standards that control for inconsistent image processing, rendering the data difficult to evaluate. Such artifacts become less of a concern as the compartments under study become larger, but even relatively large membraneless structures can display a striking degree of structural detail when examined with higher resolution (West et al. 2016; Fei et al. 2017). As this field matures, journals will need to do a better job of monitoring the image-processing pipeline to ensure unbiased image representation and a more quantitative treatment of the data.

Other commonly used assays test the importance of particular protein domains for phase separation, either through truncation/modification experiments, or through perturbing weak hydrophobic interactions by treatment with 1,6-hexandiol. Here again, while the experiments can be useful to identify important protein domains for protein–protein and protein–nucleic acid interactions which can give rise to puncta inside the cell, they are insufficient to diagnose whether these puncta arise through phase separation or through other mechanisms. Additionally, while hexanediol does disrupt some weak interactions that may lead to LLPS, it is also known to increase membrane permeability and can even cause the formation of aberrant puncta on its own (Kroschwald et al. 2017).

We briefly discussed a striking case of how using only indirect, phenotypic observations can lead to the wrong conclusion. Here, viral replication compartments formed during lytic Herpes Simplex Virus infection were shown to satisfy all of the descriptive characteristics of LLPS in vivo (McSwiggen et al. 2019). Replication compartments are round, they undergo fusion as they grow, they clearly have a different refractive index than the surrounding nucleoplasm, and they recruit many proteins that have themselves been shown to undergo LLPS in vitro (Taylor et al. 2003; Chang et al. 2011; McSwiggen et al. 2019). Given only these qualitative descriptors it would be easy to conclude that this was yet another example of a nuclear compartment generated through the generally accepted mechanisms leading to LLPS. Yet, when we performed quantitative measurements to directly assess LLPS, we were surprised to find that the replication compartments form via entirely different mechanisms. In particular, super-resolution imaging demonstrates that the compartments emerge at variable concentrations of the component molecules unlike the behavior that is predicted by a bona fide condensate phase diagram (Fig. 1), and that within each compartment the concentration of such molecules is not uniform nor randomly distributed as one would expect from a liquid state (Boeynaems et al. 2018). Moreover, using single particle tracking to follow molecules as they explore the replication compartments revealed no change in diffusion coefficient compared to the surrounding nucleoplasm, nor any evidence of an energetic barrier to entering or leaving the compartments (McSwiggen et al. 2019).

This counterexample underscores the importance of using quantitative assays that can more appropriately diagnose LLPS, rather than relying solely on descriptive ones. Unfortunately, only 14 of the 52 instances we examined reported data that could be said to be a necessary feature of LLPS (Table 1; Fig. 2C), and in only six instances was the evidence quantitative. The rest share the same descriptive criteria, but cannot be said to conclusively demonstrate LLPS in favor of other explanations, particularly in light of the example seen with replication compartments. Commonly, studies first demonstrate in vitro that a given protein is capable of undergoing LLPS. However, care should be taken when interpreting these results, as even hemoglobin and other well-folded, purified proteins can be induced to undergo LLPS in vitro given the right conditions and crowding agents (Heller et al. 1996; Galkin et al. 2002; Asherie 2004).

One cellular system in particular where current enthusiasm for LLPS has vastly outpaced the evidence is in transcription regulation mediated by enhancers, where it has been emphatically postulated by many to be dependent on a process of phase separation (Hnisz et al. 2017; Boija et al. 2018; Cho et al. 2018; Lu et al. 2018; Sabari et al. 2018; Shrinivas et al. 2018; Guo et al. 2019; Nair et al. 2019). Single-molecule experiments tracking the behavior of clusters of molecules, thought to be located at enhancers or other active DNA elements, highlight the problems of this particular interpretation (Cisse et al. 2013; Liu et al. 2014; Mir et al. 2017, 2018; Boehning et al. 2018). The observation that the clusters themselves appear and disappear with extremely short half-lives and do so heterogeneously throughout the nucleus is inconsistent with our current understanding of the formation of thermodynamically driven LLPS condensates. Indeed, transcription factor hubs in the nucleus can appear with sizes and distributions largely independent of the factor's total nuclear concentration (Mir et al. 2017), in stark contrast to the LLPS model.

While there is clearly excitement and merit in the idea that LLPS could explain long-standing questions as to how transcription factors—especially their intrinsically disordered activation domains—mechanistically drive transcription, and how this process is coordinated (Kwon et al. 2013; Hnisz et al. 2017), the evidence for LLPS formation during transcription actually occurring in cells is some of the most phenomenological. Here, in particular, the experiments that can definitively support or disprove LLPS are especially challenging. Their small size and highly dynamic nature makes them prone to misinterpretation based on morphology and their constituent molecules’ propensity to interact not only with each other, but with host genomic DNA and RNA through multiple types of interactions, makes meaningful perturbations difficult. In light of the data from herpesvirus showing that nonspecific binding to DNA can evoke many of the same descriptive behaviors, and given recent evidence that accessible DNA sites are spatially clustered in the nucleus (Xie et al. 2019), it is probable that alternative models other than LLPS can better explain the data that these studies have presented.

It is for the reasons outlined above that in our recent studies we have very purposefully avoided using the terms LLPS/condensate to describe the formation in vivo of transient local high-concentration biomolecules, in favor of the more agnostic term “hubs” (Mir et al. 2017, 2018; Boehning et al. 2018; Chong et al. 2018). This distinction is more than simply a semantic difference; as carefully outlined above, they represent distinct molecular mechanisms. Our use of the term hubs should not be construed to mean that we don't believe LLPS may be a potential mechanism for their formation. Rather, we prefer the more agnostic term precisely because we currently lack enough evidence to make definitive conclusions. Unless the biological systems represented in Table 1 can satisfy the mechanistic characteristics for LLPS condensates in vivo, along with robust evidence for functional consequences, one cannot exclude the strong possibility that the compartment in question could be forming through various cellular processes distinct from phase separation. As such, alternative models should be pursued without bias at this stage rather than treat LLPS as the null hypothesis.

## FRAP is not a test of “liquid-like” properties

**Figure 3.**
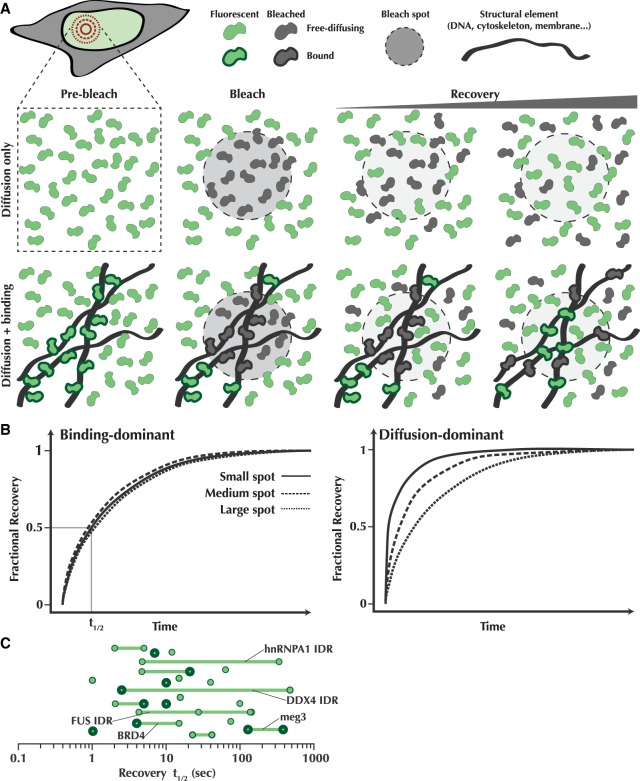
Fluorescence recovery is misleading as an assay for LLPS. (*A*) A schematic of a Fluorescence Recovery After Photobleaching experiment. Fluorescent molecules in the cell are bleached with a strong laser in one spot and the signal is allowed to recover over time. In simple diffusion, as is expected in a liquid like a phase-separated domain, mixing of bleached and unbleached molecules is only governed by diffusion. In the case where some molecules may bind to an immobile element, diffusing molecules will mix first before the bound molecules can unbind and exchange. (*B*) Binding and diffusion have different impacts on the rate of recovery and extent of signal recovery. There are many methods to analyze FRAP data, the simplest being measuring the half-life of recovery (t_1/2_). If the molecule under study has a high rate of diffusion compared to its binding rate, modulating the size of the bleach spot (dashed circles in *A*) will not affect the recovery (dashed lines). If diffusion is the limiting factor, as predicted by LLPS, then the size of the bleach spot should affect the t_1/2_ of the curve. (*C*) Reported t_1/2_ times from the studies in Table 1. Cases where the same protein or protein domain have been measured more than once are indicated by connected lines. A few such examples have been labeled for reference. Bolded circles represent measurements on endogenous proteins while the other measurements are in overexpression conditions.

Aside from the ability to undergo LLPS in vitro, our review of the literature highlighted that one of the most commonly used “gold standard” assays to diagnose a compartment as “liquid-like” is Fluorescence Recovery After Photobleaching (FRAP) (Fig. 2A). In these studies, the fluorescence recovery of a labeled protein within that compartment is assumed to imply rapid reorganization or exchange of the liquid within. As a technique, FRAP has been used extensively to measure the dynamics of protein exchange and interactions in the plasma membrane, nucleus, and specific organelles. In FRAP it is assumed that the photobleached molecules will diffuse away from the bleach spot and be replaced with new fluorescent molecules, resulting in a recovery of fluorescent signal (Fig. 3A; Sprague and McNally 2005).

Despite the prevalence of using FRAP as the “gold standard” for LLPS, there are considerable potential caveats that should be considered when interpreting the data. First and foremost, the recovery of fluorescence is not unique to freely diffusing molecules in solution. Even proteins that engage in stable, high-affinity binding interactions can show recovery (Teves et al. 2016; Hansen et al. 2017; Lawrence et al. 2018). Fluorescence recovery has a complex dependence on several parameters, including the diffusion coefficient and the concentration of the molecule being measured; the rate of its association with binding partners and their diffusion coefficients; the number and affinity of binding partners; and the technical considerations of the microscope and detectors being used (Mueller et al. 2010). Changes in any one of these parameters can influence the rate at which a bleached spot recovers. Modelling the recovery can provide quantitative insight into the underlying molecular dynamics (diffusion, binding, etc.), but it is well known that different model choices can lead to contradictory conclusions (Sprague et al. 2004; Sprague and McNally 2005; Mueller et al. 2010; Mazza et al. 2012).

It should therefore be of great concern—if ultimately unsurprising—that studies measuring FRAP in supposedly phase-separated compartments have reported subsecond (Patel et al. 2015) to minutes-long recoveries (Dine et al. 2018) for droplets generated from the very same IDR (Fig. 3). Indeed, the recovery times in the studies we examined spanned nearly three orders of magnitude, and in all cases fluorescence recovery was central to the argument that the compartment in question was phase separated (Fig. 3B).

Setting aside concerns that the range of recovery half-lives calculated spans a huge range, these one-off diffusion measurements prove little, as there are many potential biological mechanisms that may provide the same result. In-depth treatments of how binding, diffusion, and concentration affect recovery dynamics have been compiled elsewhere (Sprague and McNally 2005; Sprague et al. 2004; Mueller et al. 2010), including theoretical considerations for cases when molecules are not homogenous in solution such as in putative LLPS condensates (Sprague et al. 2006). All of which is to say that there are many physical models that can be fitted to the same fluorescence recovery curve, which makes the calculated results deeply sensitive to the chosen model. One of the major revelations that live-cell imaging has provided to biology is an appreciation for how unexpectedly dynamic molecular processes are in cells. Binding events of protein complexes that were previously expected to last in the regime of minutes to hours, based on in vitro biochemical work, actually only last for tens of seconds, even for relatively stable protein complexes (Ho et al. 2017; Teves et al. 2018). Claims of a “liquid-like rate of fluorescence recovery” (Sabari et al. 2018) therefore grossly oversimplify the potential number of models that could explain such a recovery rate.

Some groups have taken additional measures in their FRAP experiments to directly address the “liquid-like” nature of the putative compartment by partially bleaching a compartment and looking for signs of internal rearrangement, which would be suggestive of a liquid state (Patel et al. 2015). These experiments are an improvement over reporting a single recovery time, but they should still be interpreted cautiously unless control experiments are provided. One critical control, for example, is to demonstrate that the rate of recovery is dominated by diffusion rather than by binding (Sprague and McNally 2005). This can be shown by testing whether recovery is dependent on the size of the bleach spot (Fig. 3C). Further, for these experiments to be conclusive, it should be shown that the entire fluorescence signal is within the linear range of the detector, and that the recovery is only explained by internal rearrangement rather than an influx of fluorescent molecules from outside.

Recent work on FRAP specifically in LLPS systems addresses some of the above concerns, particularly with an eye to in vitro FRAP experiments (Taylor et al. 2019). Although Taylor and colleagues explicitly ignore the role of long binding events in modeling FRAP recovery—an aspect that is almost certainly not valid for many instances of LLPS in cells—they nevertheless raise many useful points. Most importantly, they show that using a bleach spot size that is similar to the size of the underlying droplet can greatly affect the resulting recovery (Taylor et al. 2019). This imposes significant restrictions on in vivo FRAP measurements, where most cellular compartments are too small to reasonably perform FRAP on because of hard physical limitations. As a result, there are instances where FRAP cannot offer meaningful insights into whether a compartment is a separate liquid phase. If recovery rates spanning nearly three orders of magnitude can all be interpreted as LLPS, then the assay becomes problematic. On its own, FRAP cannot distinguish between a separate liquid droplet and a collection of molecules generated by any number of alternate mechanisms.

## Searching for the functional significance

In the previous two sections, we have discussed how the evidence for phase separation in vivo in any given biological system is often far from conclusive. This is not to say that the compartments in question are indeed formed by a mechanism other than LLPS, but rather to highlight the significant uncertainty that still lingers. In the fullness of time, it may come to pass that some of these different putative examples of LLPS indeed turn out to be bona fide examples of phase separation. Even if this were the case, there still exists the more fundamental issue regarding functional significance of LLPS.

The observation that some cellular compartments behave like separate liquid phases has prompted speculation for a number of possible functional consequences. It has been speculated that LLPS compartments might function to facilitate cellular reactions/interactions, they may work to sequester some cellular components away from an unwanted reaction/interaction, or they may buffer the effective concentration of a given component within the cell (Bergeron-Sandoval et al. 2016; Banani et al. 2017). Briefly, the rationale behind facilitating reactions is relatively straightforward: If a select set of reactants exists at higher concentrations within a particular compartment, the reactions they perform will generally occur with much faster kinetics. The contrapositive is expected if a system is acting to sequester molecules away from a given reaction. The hypothesis that LLPS may be used to effectively buffer cells from fluctuations in cellular concentrations builds on the fact that LLPS occurs at a critical concentration, above which the solution phase separates (Oltsch et al. 2019). Thus, overproduction only results in the growth of droplets without further increasing the concentrations in either the dilute or concentrated phases, essentially providing a constant concentration of the molecule in these two compartments irrespective of the average concentration of the molecule inside the cell (Fig. 1).

Each of the above potential functions provide tantalizing explanations for how biological systems may be regulated but concrete in vivo evidence substantiating these functions in an endogenous context is lacking. Some more recent work has attempted to tie phase separation to a functional outcome (Riback et al. 2017; Du and Chen 2018; Franzmann et al. 2018; Reinkemeier et al. 2019); however, these same studies provide some of the weakest evidence that the putative phase separation process they are studying is actually occurring inside the cell, instead largely relying on biochemical experiments or previously cited work. Lacking any strong evidence for phase separation in vivo it is imprudent to imply functional effects based on the data currently available.

A recent study may help shed light on the magnitude of effects we might see from a phase-separated system. In in vitro biochemical experiments, Strulson and colleagues demonstrated that inducing LLPS resulted in a boost in the enzymatic rate of the hammerhead ribozyme, a proof of principle that phase separation can help facilitate enzymatic reactions (Strulson et al. 2012). If this principle generalizes to other types of reactions, this study is helpful in understanding what sorts of effects one might expect from compartmentalization in vivo. The authors find that the increase in enzymatic rate scales approximately proportionally with the degree of concentration (Strulson et al. 2012). While certainly in the minority, a few studies have endeavored to measure the critical concentration of an LLPS system in vivo (Berry et al. 2015; Shin et al. 2017; Bracha et al. 2018). Bracha and colleagues used ferritin “corelets” decorated with IDRs as massively multivalent over-expression constructs to robustly drive LLPS (Bracha et al. 2018). They then made precise measurements of the critical concentrations at different expression levels and valency. Their data show that the increase in concentration of the high-concentration phase is maximally around 10-fold, whereas conditions closer to physiologically relevant examples show significantly less concentration difference between the two phases (approximately threefold).

These relatively low enrichments at physiological conditions suggest a modest upper limit to the amount of reaction acceleration that can be achieved through phase separation of a single molecular species alone, though perhaps the concentration of multiple factors may yield additional acceleration. Recent evidence in cells supports such a modest limit: Two halves of a reaction targeted through in vivo overexpression into droplets yielded less than a twofold increase in the reaction selectivity and simultaneously a marked decrease in reaction efficiency (Reinkemeier et al. 2019). Only by further promoting association through the addition of kinesin motor domains to spatially concentrate their reaction could synergistic improvements of five- to 10-fold be achieved (Reinkemeier et al. 2019). This is not to suggest that small changes in concentration cannot have important phenotypic outcomes, but it is important to keep in mind these upper bounds when considering functional implications; and particularly when those functional implications are speculative in nature.

Current data present a similarly modest picture when considering how effective LLPS might be at sequestering a given molecule away from unwanted reactants. Because LLPS is intimately tied to the critical concentration at which a droplet forms, we can use the concentration of the dilute phase to estimate the degree to which LLPS improves protein sequestration. Again, using the FUS corelet system as an extreme example, the difference in concentration of the corelets before and after induction of LLPS in the dilute phase is modest, perhaps twofold at most (Bracha et al. 2018). For such a system to be an effective and meaningful mode of regulation, it would need to be sequestering molecules that are exquisitely sensitive to component concentration. It is of course possible that such a system exists, but these limitations should be explicitly considered when proposing phase separation as functionally relevant for sequestering reactants.

The above points suggest that the effects of LLPS on either facilitating or sequestering reactions will likely be quite subtle, and difficult to adequately test, particularly in a physiologically relevant concentration regime. The hypothesis that phase separation serves as a means to buffer biomolecules is equally challenging to verify. One may speculate on whether there is evidence that evolution has selected for optimal LLPS behavior under a given set of conditions, but there is still too little data to begin to address these types of questions. Until clear, testable predictions are made and investigated in vivo under physiologically relevant conditions, the functional consequences of phase separation will remain shrouded in uncertainty.

## Finding a path forward

The notion that cells have evolved to use liquid–liquid phase separation as a means of further compartmentalizing the intracellular environment to specifically regulate biochemical reactions is a compelling one. We do not wish to suggest that phase separation can never happen inside the cell, nor that phase separation is inconsequential to certain cellular functions. To be sure, there are clearly examples where LLPS remains the most suitable interpretation of the current evidence. Rather, with the research community so intoxicated by the current crop of studies and the tantalizing promise to explain the mechanistic underpinnings of subcellular organization, it is also important to recognize the potential for other explanations and the current lack of concrete evidence to point to one interpretation or another.

It may be the case that LLPS is a pervasive phenomenon in subcellular organization, mediated by multivalent interactions through intrinsically disordered protein domains, RNA, or DNA molecules. It may also be that the various cellular systems proposed to phase separate will still stand up to greater scrutiny and to assays that can more faithfully diagnose LLPS. These assays should directly probe how the compartment responds to changes in molecular concentration, binding-interaction strength, temperature, and study the effect of putative LLPS on the compartment in question's constituents. However, in the absence of these more robust data, LLPS should not be invoked as the more likely interpretation of otherwise phenomenological observations, and alternative hypotheses should be formulated and tested to provide real biological insights.

In order to advance the field as a whole, it is clear that better assays and cellular systems are needed. Unfortunately, there is unlikely to be a one-size-fits-all suite of assays that can probe LLPS, and experiments will need to be thoughtfully tailored to the system at hand. An important first step, given that LLPS is intrinsically tied to cellular protein concentration, is a concerted effort to move away from experiments that overexpress proteins likely to participate in LLPS, even if only to a small degree. Instead, it is worth the time and effort to tag the molecule under study in the native genomic locus to ensure endogenous levels of expression and protein concentrations. It is also clear that roundness and the ability to fuse are not sufficient evidence, and similarly FRAP experiments, if used, must be held to a higher standard than they are currently and the results interpreted with caution.

Better and more creative assays are in high demand. While the appropriate experiments will clearly depend on the exact system under study, there are at least a few promising avenues. Advances in light microscopy and spectroscopy allow quantitative measurements of absolute protein abundance, with and without fluorescent labels (Wang et al. 2011; Mir et al. 2012; Wei et al. 2017; Cai et al. 2018; Walther et al. 2018). For example, even if the exact critical concentration remains elusive to quantify for a given system, theory would predict that within a cell, the putative condensates should have equivalent concentrations of the phase separation molecule (as assayed by the fluorescence intensity per volume, for example). Single molecule tracking experiments would be a desirable substitute for FRAP and have proven to be critical in uncovering an alternative compartmentalization mechanism in the case of herpesvirus (McSwiggen et al. 2019). These results suggest that the application of single-particle tracking techniques in other systems may prove fruitful for examining the effects of putative phases on molecular behavior, as would be predicted by theories around viscoelastic materials (Elbaum-Garfinkle et al. 2015; Wei et al. 2017; Niewidok et al. 2018).

Another strategy that may more directly diagnose in vivo LLPS would be acute depletion using endogenously appended degron tags (Nishimura et al. 2009), which should allow one to follow the degradation of proteins to determine whether compartments follow the types of behaviors that LLPS would predict (Fig. 1). Other microscopy approaches such as localization of individual molecules within the compartment (Freeman Rosenzweig et al. 2017; Narayanan et al. 2019) or super-resolution imaging analysis of compartments which reveal fine structure (West et al. 2016; Fei et al. 2017) may help in testing LLPS as a model, as well as its functional consequences. Combined with single-particle tracking experiments, these and other assays might reveal the specific concentration- and state-dependent manner that LLPS predicts, as well as effects on the molecules involved such as changes in diffusive behavior or energetic penalties for crossing between one phase to another.

Lastly, the ability of a protein to undergo phase separation when purified in vitro is an important finding to understand intrinsic properties of that specific protein, but these simplified systems cannot faithfully recapitulate the richness and complexity of interactions that occur within living cells. While these experiments are very useful for defining critical reaction partners, modifications, and energetic parameters, appropriate caution should be exercised when drawing equivalencies between these reconstituted conditions and the environment of the cell in vivo. A protein may phase separate in a test tube, and when produced at extreme quantities may also undergo LLPS inside the cell, but perhaps the more interesting and physiologically relevant interactions are found in less extreme conditions. It should be encouraged for future studies to include a more nuanced discussion on alternative models to phase that will likely provide valuable new insights.

## Conclusion

Phase separation as an organizing principle in biology has compelled us to revisit old ideas in a new light and will likely continue to do so. As we have shown, the current state of the field is rich in descriptive evidence for phase separation in cells, but in most cases lacks crucial conclusive data. Roundness, fission and fusion, and speedy fluorescence recovery may bolster support for phase separation as a model—when proper controls are also provided—but the existence of counterexamples that share these properties in the absence of LLPS emphasizes the need for more rigorous and quantitative examination in cells with proteins expressed at the endogenous level. Further, experiments demonstrating the functional impact of phase separation, both at the phenotypic and mechanistic level, remain sorely lacking. Whether or not LLPS turns out to be a general phenomenon of broad functional utility, it should be appreciated that the formation of condensates likely represents only one of many potential avenues that the cell can use to organize its contents to facilitate critical biomolecular interactions at the right scale and temporal cadence.
